# Integration of artificial intelligence and precision oncology in Latin America

**DOI:** 10.3389/fmedt.2022.1007822

**Published:** 2022-10-13

**Authors:** Liliana Sussman, Juan Esteban Garcia-Robledo, Camila Ordóñez-Reyes, Yency Forero, Andrés F. Mosquera, Alejandro Ruíz-Patiño, Diego F. Chamorro, Andrés F. Cardona

**Affiliations:** ^1^ Department of Neurology, Fundación Universitaria de Ciencias de la Salud, Bogotá, Colombia; ^2^ Foundation for Clinical and Applied Cancer Research – FICMAC, Bogotá, Colombia; ^3^ Division of Hematology/Oncology, Mayo Clinic, Scottsdale, AZ, United States; ^4^ Molecular Oncology and Biology Systems Research Group (Fox-G), Universidad el Bosque, Bogotá, Colombia; ^5^ Direction of Research, Science and Education, Luis Carlos Sarmiento Angulo Cancer Treatment and Research Center (CTIC), Bogotá, Colombia

**Keywords:** artificial intelligence, Latin America, oncology, technology, precision oncology

## Abstract

Next-generation medicine encompasses different concepts related to healthcare models and technological developments. In Latin America and the Caribbean, healthcare systems are quite different between countries, and cancer control is known to be insufficient and inefficient considering socioeconomically discrepancies. Despite advancements in knowledge about the biology of different oncological diseases, the disease remains a challenge in terms of diagnosis, treatment, and prognosis for clinicians and researchers. With the development of molecular biology, better diagnosis methods, and therapeutic tools in the last years, artificial intelligence (AI) has become important, because it could improve different clinical scenarios: predicting clinically relevant parameters, cancer diagnosis, cancer research, and accelerating the growth of personalized medicine. The incorporation of AI represents an important challenge in terms of diagnosis, treatment, and prognosis for clinicians and researchers in cancer care. Therefore, some studies about AI in Latin America and the Caribbean are being conducted with the aim to improve the performance of AI in those countries. This review introduces AI in cancer care in Latin America and the Caribbean, and the advantages and promising results that it has shown in this socio-demographic context.

## Introduction

Despite advancements in knowledge about the biology of the different oncological diseases, complex interactions between the tumor and the microenvironment, the cellular heterogeneity, and Darwinian evolution of tumors; it continues to be a big challenge in terms of diagnosis, treatment, and prognosis for clinicians and researchers (1). To solve this complexity, technologies such as Artificial Intelligence (AI) have continued to develop. This computer science-derived technology encompasses a wide variety of potential functions that could improve different aspects like predicting clinical or molecular parameters for prognosis, cancer diagnosis, cancer research, and personalized medicine (2).

AI, a term coined in the 1950s, is defined as a machine's ability to learn, recognize and relate patterns for decision-making. AI use has increased in medical oncology over the last decade, reflecting the major impact that computer systems and artificial neural networks can have. Inside this kind of technology, we can find Machine learning (ML) derived from AI and Deep Learning (DL) a subfield of ML (3). The first one is based on the performance of mathematical and statistical operations inside artificial neural networks that have the potential to transmit signals between them. That information transmission allows encoding and decoding of data. These ML models can be supervised or unsupervised. The second one represents a more recent technique with better performance compared with previous AI algorithms in different disciplines, related to complex inputs analysis using complex structures (neural networks) which gives it a new potential for the resolution of some of the more complex problems of oncology, like cancer image analysis radiological and digital pathology included (4). Those systems simulate human data processing and sometimes overcome it in certain tasks. Based on a large and diverse set of information collected through minimally invasive techniques, AI has shown an unquestionable potential for care and impact on the path of early cancer detection, risk control, relapse, and guidance of therapeutic options (3).

The amount of new literature in these areas is undeniable and points to the widespread and possible applicability of cancer solutions and precision medicine (5). However, although AI has provided a suitable solution to some drawbacks, its implementation has some intrinsic problems. These problems include data standardization, interpretability, validation and utility, integration of these technologies with healthcare professionals, and regulatory and legal issues, among others (6). Other issues correspond to cancer-related challenges that are not easily resolved by these technologies and require an updating process (ex. Reinforcing algorithms) to exploit clinically feasible utility (7). Finally, some additional disparities in the research and execution are prevalent and more evident in low and medium-income countries (LMIC), such as Latin America, due to socio-economic imbalances which can affect funding for critical areas for the development and integration of these models (8).

### Impact of precision oncology in cancer care

With the development of molecular biology, diagnosis, and therapeutic tools in the last few years, the paradigm of “one-size-fits-all” therapies for malignant neoplasms has been completely revoked. Precision oncology (PO) is one of the reasons for that change in the therapeutic approach. PO involves a series of diverse strategies that try to understand the biology and pathophysiology of patient-specific malignancy (9). The genomic landscape of cancer cells is the focus of PO, but not the only one. Understanding the complex genomic interactions of malignant cells has been used as the base for the development of targeted therapies, and these therapies have become the new and personalized approach for oncologic patients.

However, several previous requirements must be fulfilled to finally select the right and precise therapy: correct diagnosis, molecular profiling, and proper staging. All these variables represent data (sometimes complex). That's when AI can play an important role. Integration of clinical and genomic information can be challenging, especially, when specific data of genomic characteristics are present to the clinician and an important number of therapeutic options could be considered (10).

Although the implementation of PO can be challenging due to the costs of molecular techniques and diagnosis tools, it has a significant effect on oncological patients, improving survival, life quality, and optimizing therapy. For example, one study by Quinn et al., found that among one thousand patients with different oncologic diagnoses, 25% showed a partial response rate to treatment and almost 18% presented a stable disease (11). The impact of PO on patient outcomes is promising and it could be the next guide for decision-making in a clinical context. Also, Haslem et. al, analyzed nearly 1,800 patients and their data showed a reduction of almost 7% reduction in general costs in the last three months of life among patients who received targeted therapy (12). Here, the study suggests that PO could improve not only survival in oncologic disease, but also optimize the healthcare costs among these patients, both important variables for the healthcare system.

### Artificial intelligence and precision oncology

The development of advanced informatic tools, the progressive improvement in genomic sequencing, and related technologies have allowed a constant growth in the body of information that we can obtain from patients (genomics, transcriptomics, proteomics, metabolomics, and epigenomics). We face big obstacles managing, organizing, and processing the vastness of these data, but also encounter great opportunities as this information will be valuable for a better understanding of patient populations in a more personalized and precise way (13). AI is a set of informatic algorithms and computing frameworks that are usually used to simulate, enhance, and or even replace human reasoning in the form of decision-making, speech recognition, language understanding, visual perception, and pattern recognition (14). The final goal of using AI in PO is to accomplish tasks that would normally be performed by human experts in a certain field. However, AI and the rising computing power might sometimes perform these tasks more efficiently, especially in cases in which big data is involved and information processing becomes overwhelming for a human. The power of AI will not only be able to manage and process these data but also to find novel and interesting associations between multiple variables. In this section, we will discuss the role of AI in the oncology clinical practice, touching on different topics involved in the diagnosis and treatment of malignant conditions.

### Molecular profiling

**Figure 1:**
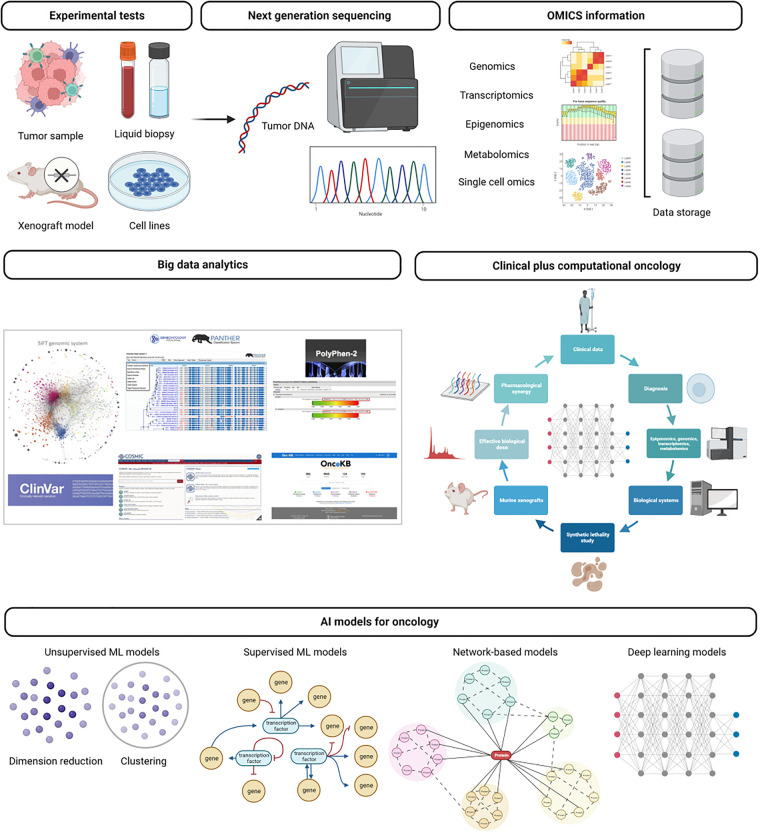
General application of artificial intelligence to genomics. Next-generation sequencing (NGS) data comes from genomics, transcriptomics, epigenomics, or single-cell omics. These approaches led to an accumulation of large-scale datasets to solve challenges of cancer research, molecular characterization, tumor heterogeneity, and drug target discovery. As the large scale of omics data accumulated, various machine learning techniques, including graph algorithms and deep neural networks, are applied for dimensionality reduction, clustering, or classification.

Neoplastic conditions are highly heterogeneous. Advancements in molecular and cellular biology have shown that tumors from the same tissues are not necessarily identical. Molecular profiling uses techniques related to NGS, like whole genome sequencing and RNA sequencing (which can even be done at a single-cell level), immunohistochemistry, and epigenomic studies, to determine different presentations of a specific tumor. Molecular profiling better characterizes subsets of patients with specific genetic, transcriptomic, metabolic, and epigenomic patterns (10, 15) (Figure 1).

Initially, molecular profiling was performed at a small scale, and analysis of outcomes related to certain disease molecular patterns was extracted from clinical studies usually with a small sample of patients. One example that might illustrate this idea is the case of breast cancer (BC), in which initial molecular profiling was restricted to the expression of certain proteomic markers involved in BC pathogenesis like estrogen receptors, progesterone receptors, and the upregulation of HER2/ERBB2 (16, 17). This initial approach classified BC according to the presence of one or more of these traits. Each subtype was analyzed for outcome prognoses like overall survival, risk of recurrence, and treatment efficacy.

Nowadays, BC subtypes have been furtherly characterized, with more subtypes present in each category, creating subgroups of patients that might benefit from interventions and subsets of patients with different outcomes risks. The Cancer Genome Atlas (TCGA) has played a critical role in these developments. The TCGA is an international effort of scientists and clinicians gathering immense amounts of data from cancer patients, including clinical, histologic, environmental, and omics data. Since its inception, the TCGA has produced approximately 2.5 petabytes of data. All this data is publicly available for researchers in the general public, to use and analyze to find new patterns of disease that might precisely impact cancer outcomes (18).

Multiple groups of researchers have used AI, especially ML algorithms, and data mining approaches (19) to elucidate valuable meanings from all these data, with different applications in cancer research and clinical practice (19). Van IJzendoorn et al*.* used machine learning algorithms to analyze transcriptomic profiles from soft tissue sarcoma samples and run deep neural networks to successfully find novel prognoses, diagnostic markers, and possible treatment targets (20). Abraham et al*.* used a very interesting approach. They analyzed 77,044 genomic and transcriptomic profiles and used them to evaluate samples from patients with cancer of unknown primary origin, accurately identifying 71.7% of cases. Its precision was even higher than that of pathologists, who needed to change the diagnosis in 41.3% of cases (21). Like these groups, there are multiple other approaches available that are helping refine precision medicine (22).

### Germline variant discovery

The discovery of germline variants is a revolutionary pipeline in bioinformatics (23). The main feature of germline variant discovery is based on comparing a species normal genomic sequence with sequences obtained from a patient using NGS techniques. Therefore, mutations and genetic alterations can be found in certain genes and non-coding regulatory regions by comparing a cancer genome with a template of healthy DNA. This process of comparing and identifying differences is known as variant calling (14). The workflow for variant calling is a somewhat bothersome process in which a lot of bias and propensity to error might be found, not to mention high time consumption. Some steps include improving the data quality by removing duplicates, insertions, deletions, sequence re-aligning, and base recalibration. AI can help perform all these pipelines with increased accuracy and higher efficacy. Kothen-Hill et al*.* successfully performed variant calling from liquid biopsies, detecting early-stage lung cancer (24). These methods have been continually improved. Wood et al*.* developed a ML model that successfully calls cancer-related variants with a sensitivity of 97% and a predictive value of 92% using exosome data from the TCGA (25). Beyond variant calling, AI is also being used to predict the impact of new mutations in the expression, function, and structure of their translated transcripts. This is done by using algorithms that perform homology modeling. Some models can even predict if a new mutation is prone to initiate a neoplastic cascade by analyzing the position in the coding region in which the mutation is located (26).

### Imaging genomics (Radiomics/Radiogenomics)

**Figure 2:**
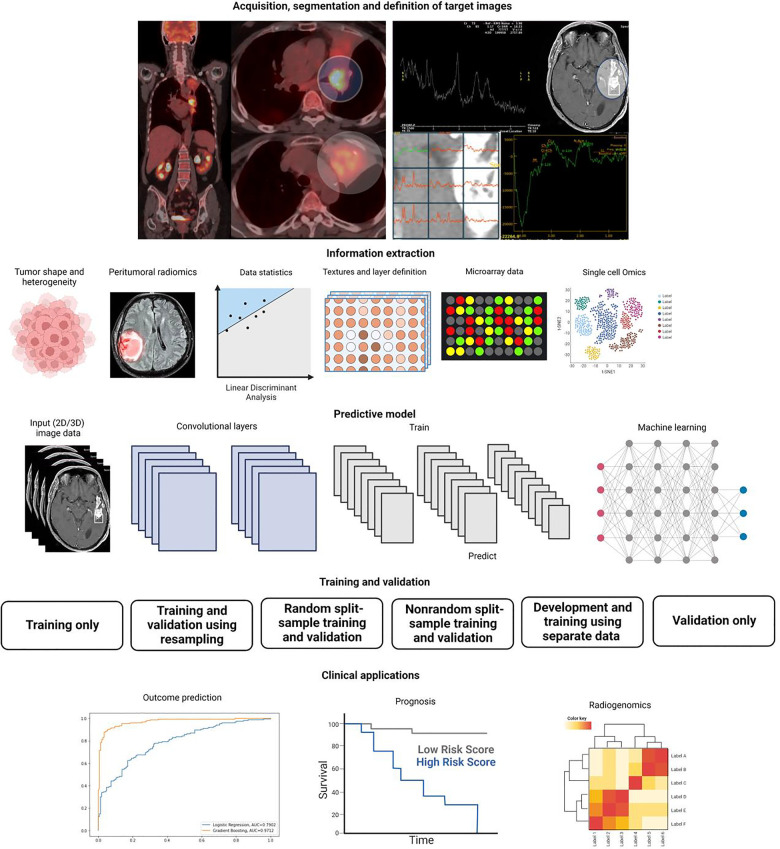
Artificial intelligence and radiomics.

The association between a patient's tumor imaging and genomic data product of NGS is known as radiomics or radiogenomics (27). Pattern recognition abilities from AI algorithms are currently in use for the analysis of different images like histologic or radiographic pictures. AI can identify structures, shapes, lines, points, colors, and boundaries to differentiate healthy tissue from tumor images. Currently, various AI models can outperform medical imaging experts in the diagnosis of cancer and in making prognostic predictions (28) (Figure 2).

Furthermore, information derived from the analysis of many number of images can be associated with certain traits obtained during molecular profiling. Thus, molecular classification and sometimes prognosis can be predicted from the analysis of diagnostic imaging (28). In 2022, Yin et al*.* published their data on the use of a convolutional network to assess the presence of brain metastases vs the analysis performed by senior and junior radiologists. The model was able to predict the presence of metastasis with higher accuracy than the radiologists. These kinds of studies have also been performed also in different tumors like mantle cell lymphoma, to predict prognosis and treatment response rates (29). Today the field of radiogenomics is in development, but it promises to become a very powerful tool for oncology clinicians and surgical specialists.

### Digital pathology

Initially considered the process of digitalizing images from slides for further analysis, digital pathology has transformed into a discipline in which computational methods and AI are at the vanguard. Like radiogenomics, digital pathology provides the framework for using ML models in the diagnosis of multiple conditions. Cancer is one of the most studied pathologies in this field. With the increased incidence of malignant diseases in our populations, refinement in diagnostic pathology methods and classifications is needed. Sometimes it can take a pathologist a considerable amount of time to give a proper diagnosis with adequate risk stratification, presence of determined biomarkers, molecular profiling, and proper tissue processing. This amount of time dedicated to analyzing samples plus the increased number of samples needed to be analyzed creates a delay in diagnosis or even induces pathologists to perform these processes as fast as possible, which increases the risk for error, a common human trait. AI in digital pathology has been used to perform analysis related to TNM staging (30). Analysis of immunohistochemistry, which is common in oncology diagnosis have also been studied using AI models for expression of PD-L1 (a trending biomarker to determine candidates for immune checkpoint inhibition) and determining hormonal receptor status in BC (22).

In general, digital pathology, radiogenomics, and molecular profiling create a myriad of tools that when analyzed by a well-trained AI model, can deliver valuable results that can be applied to PO. The era of information is certainly expected to improve our understanding of cancer, who should be treated, and how it should be treated.

### Artificial intelligence and cancer treatment

Cancer is a complex etiology, as its molecular characteristics and interactions are too. In the last decades, biomarkers have become a cornerstone for cancer treatment for their many implications: risk assessment, screening, differential diagnosis, classification and cancer typification, response to treatment monitoring, and prognosis in general (14, 31). These biomarkers can be identified by several techniques focused on the detection of genomic alterations. Next-generation sequencing (NGS) performed by invasive and non-invasive methods, like liquid biopsy, can identify genetic alterations that might suggest the selection of one therapy, the escalation of another, or the de-escalation of another one (32).

However, genetic material is not only the source for biomarker discovery. Proteomics and analysis of specific amino acid sequences can also offer a new approach for biomarkers. Proteins represent the result of genetic material engineering inside the cell. Machine-based proteomics uses mass-spectrometry and deep learning to build a data network based on peptides to identify common synthetized proteins related to a specific oncologic disease (19, 33). It is interesting that other characteristics such as imaging findings or radiologic patterns can serve as biomarkers today. They could be used to construct prediction models with the potential to generate data about prognosis or prediction for patients. Specific textures, forms, borders, or intensity under imageology techniques could be analyzed by AI and associated with a unique diagnosis (34).

On the other hand, biomarkers can also be targets for a therapeutic approach. Commonly, for oncology drug development, as for other drugs, the process begins with target identification, followed by lead optimization, pre-clinical development, clinical development (Phase 1, 2, and 3 studies), and regulatory approval (35). This process usually takes months, or even years, and complications can occur in every phase. AI has the potential for the prediction of function, differences, and weaknesses of molecules derived from the transcriptomics database to identify a new therapeutic target (36). ML biology analysis can shorten the process of target identification modulating complex information related to the heterogeneity of molecules and biochemical interactions. Genomics and proteomics can be used for drug discovery. Identifying receptors, microRNAs, transcription factors, inhibitors, co-effectors, and other molecules have been analyzed for target therapy (37, 38).

### Bias, legal, and ethical issues in cancer-related artificial intelligence

In the case of cancer care, the application of AI includes cancer detection, characterization of the patient's prognosis, and optimizing care or clinical operations by increasing the health system's capacity and relocating resources. However, it is important to consider the legal and ethical problems that society faces due to the use of AI. These include respect for privacy, the need for surveillance mechanisms, the prevention of secondary errors because of gaps in knowledge/information, and the prevention of errors in procedures (9).

#### Legal risk

The European Parliament Resolution was created, supervised, and published by the department for “*constitutional issues and rights for citizens*” in response to the request from the European Parliament committee on legal issues. This report focuses on the critical need to create a resolution to safeguard the interests of human beings by legislating the use of robots and AI in daily medical practice and clinical studies (9).

Currently, it is still a topic of debate whether AI fits all legal categories or new categories must be developed to make clinical practice more transparent, equitable, fair, and respectful (9, 39). The use of informed consent for data use, security, inclusion, transparency, fairness in the algorithms and confidentiality are essential factors to be considered from the legal perspective. Since algorithms are not ethically neutral, their results must reflect human ethical standards, either good or bad. For example, in cancer screening programs, a learning algorithm can prioritize minimizing false negatives rather than minimizing false positives or acting differently depending on the characteristics of the affected tissue or different sociodemographic groups (39). AI can create “a black hole,” defining the most important: interpretability. If the algorithm explains itself, humans can know what it is doing and what variables it encodes. So, in theory, all AI systems will inevitably reflect numbers (patient outcomes), and these values will be challenging to discern (undetected systematic errors) in large populations (40, 41).

The introduction of AI in the health system is exponential, without clear regulators and established monitoring processes or patterns, meaning that we do not know what legal responsibility AI has during unsuccessful events. This “regulatory vacuum” in the application of AI creates the need to build a new regulatory framework to protect vulnerable patients, perform realistic identification and maintain (42).

#### Bias

Evidence suggests that AI models generate biases. However, one should never forget that underlying information rather than the algorithm is responsible for these biases. The AI models reflect information that contains human decisions or information that reflects second-order effects of social pressure or historical inequities. Depending on how the information is used and how it is collected, it can also contribute to biases (training information or algorithm designs) since it is possible that information-generating systems could create repetition feedback loops, leading to errors in the results and outcomes (43).

It is well known that health systems and evidence-based medicine are biased towards disadvantaged groups since those groups are under-represented in the databases. The predictive outcomes from training models must be carefully selected to ensure that they are not associated with socioeconomic biases, resulting from inequities in the health system. Automation biases can also occur when humans accept the machines' decisions even if the result is wrong. Clinical expertise and correlating physical examination and the patient's medical records are essential to prevent those biases. If the healthcare professional skips those steps, it will result in more biases that will lead to less cost-effectiveness and overdiagnosis in those cases (42).

AI systems require a significant quantity of high-quality information for training, validation, authorship, consent to use, and protection of critical information. Therefore, some technology companies are now offering software for data collection or history recording systems that were created to be used exclusively for patient/clinic medical records. However, concerns about confidentiality are still a critical point, and questions about the ownership (software companies vs. patients) of the data collected in those systems are still on the table (44).

In addition, another major issue is the transferability of algorithms to all existing platforms. Algorithms are created and tested in specific environments; therefore, they will not automatically work in another environment. For this reason, efficient and effective transfer training is mandatory to avoid the above-mentioned weakness.

#### Medical ethics and responsibility

The primary concern of clinicians, particularly those who diagnose patients, is the exclusion of their participation in the clinical process. That's why merging human medical knowledge and AI is imperative, leading to more efficient and effective medical care.

Additionally, with the introduction of AI, it is possible that we could face a disruption of the traditional conception of medical responsibility over patients. It is important to keep in mind that patients should always have a personal relationship with their treating physician, even if AI is being used as a supporting tool during the clinical process. Additionally, healthcare professionals should always take responsibility for treatment decisions, considering the patient's medical record, physical examination, and medical expertise; AI should only be considered as a supporting tool and should never replace healthcare professional knowledge (39, 42, 44, 39–44) So, if physicians only rely upon AI, the ability to diagnose, think, analyze and develop necessary clinical skills for daily medical practice could be hindered (39–44).

Currently, there are no recommendations or regulations for legal or ethical issues regarding the use of AI. Therefore, the integration of AI into daily clinical practice and research is still a challenge.

### Artificial intelligence in oncology: perspective for developing countries

Currently, cancer is considered the second cause of death in Latin America and the Caribbean and approximately 600.000 people die from cancer annually in the United States (US). Additionally, each year 1.8 million people are diagnosed with cancer in the United States and more than 1.3 million people in Latin America and the Caribbean (45, 8). To provide optimal cancer care a specialized multidisciplinary team in continuous update and multiple special facilities are required. In addition to that cancer care is costly, it is reported that in the US the expenses for cancer treatment in 2020 reached 173 billion dollars (6).

**Figure 3:**
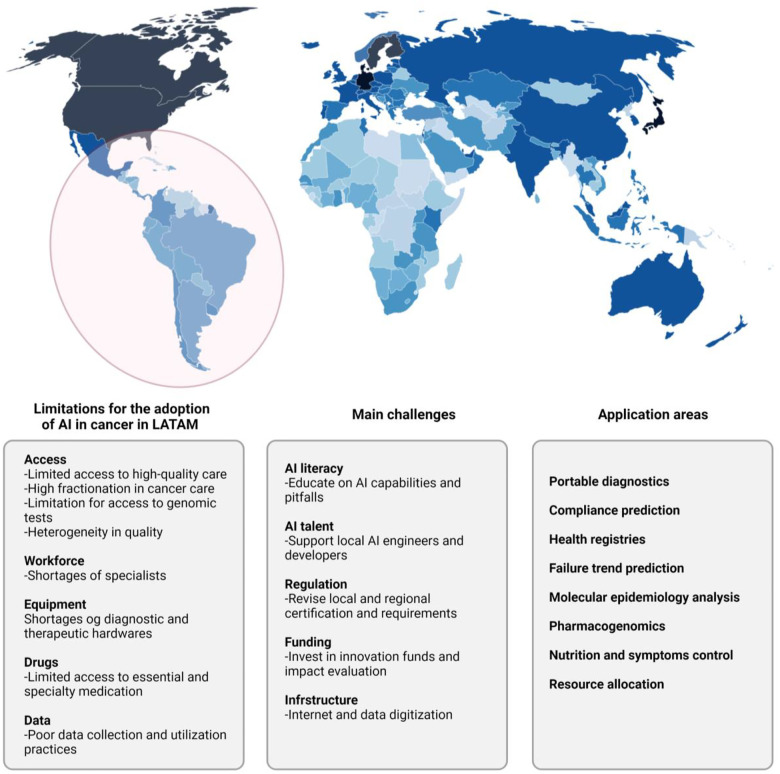
Integrating AI to cancer genomics in Latin America and Low Middle-Income Countries (LMICs). AI technologies are forecast to add US$15 trillion to the global economy by 2030. According to the findings of our Index and as might be expected, the governments of countries in the Global North are better placed to take advantage of these gains than those in the Global South. There is a risk, therefore, that countries in the Global South could be left behind by the so-called fourth industrial revolution. Not only will they not reap the potential benefits of AI, but there is also the danger that unequal implementation widens global inequalities. In 2017, Oxford Insights created the world's first AI Readiness Index.

It is known that cancer control in Latin America and the Caribbean is insufficient and inefficient, considering the previously mentioned characteristics. It is known that there are major limitations to access to trained healthcare professionals, new therapies, and adequate facilities for cancer care, because of inadequately distributed budget al.locations, and geographical and cultural barriers (8) (Figure 3).

Therefore, some studies about AI that have been conducted in developing countries aim to improve the performance of health facilities, resource allocation from a systems perspective, reduction of traffic-related injuries, and other health system issues with cost-effective interventions (46, 47). To date, some of the most promising results regarding AI are represented in disease diagnosis, specifically digital pathology, radiographic imaging, and clinical photographs (48). Most studies regarding AI in oncology conducted in developing countries were about disease detection in cervical cancer and pre-cervical by microscopy or cervigrams; an accuracy of greater than 90% has been reported (46). Preliminary results in this area are promising and AI could be considered a very good option in countries with a lack of health providers, AI could help to supplement clinical knowledge (49).

Despite the encouraging results, some aspects needed to reach successful results with AI in oncology are limited. For example, personnel training to be qualified to register, collect, and interpret data is difficult in Latin America and the Caribbean because of the geographical limitations and the fragmented data capture due to the several healthcare organizations or insurance providers (47, 50, 51). Furthermore, the lack of open access structured datasets that integrate phenomics, genomic, and environmental health determinants is another imitation of AI implementation (31, 19). To reach this goal new types of data sharing protocols and standards on inter-operability and data labeling are needed; at the moment this is only happening in the UK and Europe (19). In addition, the successful AI methods used in current studies, don't consider general health system costs or psychological or social consequences (46).

AI promotes equity in patient access to high-quality health care and stimulates investments in machine learning and data science, leading to scientific and economic development. However, to reach optimal cancer care with help of AI in developing countries further studies are needed that consider local needs, health system constraints, and disease burden of the location rather than the availability of data and funding.

### Challenges and limitations

Although AI and PO are showing promising results for the future of oncology, this path is full of interesting challenges and some limitations intrinsic to the complexity of these technologies and others related to the implementation of these in Low and Middle-income countries (LMIC) (Table 1).

**Table 1:** Limitations for AI in cancer care in LMICs.

Type of limitation	Description	Strategies to overcome the limitation
Data Standardization	Difficulties with data collection	Healthcare professionals training and economic resources for technology.
	Data heterogeneity	Strategies such as mCODE, PROM, HistoQC, Deep Focus, or GAN-based image generators are being developed.
Interpretability	Difficulties with outcomes interpretation	Some models to ease the interpretation of the results are being developed
Validation and Utility	Difficulties with AI validation on real clinical scenarios	More studies are being conducted

#### Data standardization

Data from healthcare institutions often are not homogenized; on the contrary, tend to be disorganized, and sometimes data are absent. Data quality is critical since current AI models require the standardization of terminology and data collection for better performance. AI algorithms were created to be used primarily with a particular system; if the algorithm is used with other systems it would probably show low performance. This represents a striking barrier because these data storage systems are not generally available in all healthcare facilities (52).

Radiomics and digital pathology are two fields where standardization is vital and more complex. Images are widely dispersed in different hospitals or healthcare centers, sometimes the images depend upon the operator, and sometimes the image known as the gold standard of a certain disease isn't even performed, like in the case of tumor segmentation. This heterogeneity complicates the comparison between images and represents a barrier for AI.

In the case of digital pathology, high-quality data are essential for the correct functioning of AI algorithms. For instance, in AI-mediated segmentation of a specific structure in a whole slide image (WSI), the completion of this task highly depends on the veridical reference annotations (data) by expert pathologists, therefore the need for well-structured and homogeneous organized data. Nevertheless, it is known that the current absence of this standardization concerning staining reagents, protocols, and section thicknesses (of radiologic images) is reported (52–30). To address these issues there have been some initiatives such as the minimum Common Oncology Data Elements (mCODE), which tries to identify and standardize essential cancer data in electronic health records (EHR). Another method of standardization currently being used is the Patient-reported outcome measures (PROMs), which consists of early data collection directly from the patient. In the case of radiomics and digital pathology, some automated tools such as HistoQC, Deep Focus, and GAN-based image generators are being developed to help this cause. Implementing some of these solutions although effective carries an additional burden on healthcare professionals putting them at additional risk of burnout (6, 53).

#### Interpretability

One of the main criticisms of AI and deep learning (DL) has been the interpretability of the output, gaining the name of “black box” due to the difficulty in the interpretation. These technologies require servers and trained staff in bioinformatics. This has caused a steeper clinical adoption curve of this technology by oncologists (54, 55). Due to this fact, some models such as *post hoc* methods or supervised ML have been studied to facilitate the interpretation of the output once DL made its prediction. One example of the utilization of these was exemplified in a study of brain tumors by tumor biopsy specimens, where to one participant with a high-grade tumor a heat-map was performed indicating areas of early microvascular proliferation (an indicator of progression); facilitating interpretability (30). Another example was the combination of DL and hand-crafted models to increase interpretability in the study by Wang et al. where DL was used first for nuclear segmentation and posteriorly hand-crafted was performed for investigating nuclear shape and texture in digital histologic images of early-stage non-small cell lung cancer (56). Although these strategies for enhancing interpretability are helpful and probably more applicable in real clinical scenarios, these technologies have been criticized because DL should not require additional models to facilitate its interpretation (57).

#### Validation and utility

The advances in AI and PO are notorious and the literature on DL algorithms performance is extensive, however, the validation of these technologies in real clinical scenarios remains troublesome. To achieve this end, these models must be internally and externally validated, and prospective studies or prospective-retrospective analyses should be used (40). A fair number of studies are internally validated comparing the AI model to the expected results (thrown by the gold standard) relying on parameters such as sensitivity, specificity, and Area Under the Curve (AUC). These previous give a landscape of AI performance, regardless, few amounts of studies utilize external validation, therefore, forcing a cautious interpretation of performance due to no protection to specific characteristics of one cohort that could overfit AI performance. Beyond validation, the clinical utility requires additional studies to investigate specific clinical endpoints, such as overall survival, progression-free survival, toxicity reduction, and improved quality of life, among other objective response rate measures. Although the gold standard of these additional studies is a randomized control trial, the intrinsic complexity of carrying out these studies has opened the door for the applicability of prospective-retrospective analysis to solve this issue (52, 54).

#### LATAM challenges and limitations

In addition to the previously discussed, LMIC have other unique situations concerning limitations and challenges. Precision medicine has not been a field with extensive research, with less information available in areas with high population and ethnic diversity. Deficiencies in certain areas make this task even more challenging, such as inadequate infrastructure, insufficient resources, suboptimal regulations, and scientists not at the forefront of these technologies (58). With respect to PO in Latin America, although it has gained interest, as demonstrated by LMIC being the area with the most significant average annual growth from 2005 to 2012 in clinical trial participation (33%). The design of this type of studies that requires molecular-based selection criteria, bioinformaticians and specialized personnel, biotechnology companies, and government regulatory measures. All of that requirements added to socioeconomic disparities intrinsic to Latin America and accentuated by differences in the public and private healthcare sectors, making the implementation of AI a complicated task in Latin America, sometimes requiring multicenter and multinational research with the aim of more available data (40, 59, 60).

## Conclusion

It is known that the use of AI as a support tool in cancer care has shown auspicious results. Currently, some studies and strategies are being conducted in Latin America and the rest of the world to incorporate AI into daily clinical practice in cancer care. However, more clinical trials and more sociodemographic and economic studies are needed to consider the use of AI in the standard clinical practice in Latin America, a challenging scenario for technology innovation.
